# The role of sociodemographic and clinical factors in the initiation and discontinuation of attention deficit hyperactivity disorder medication among young adults in Sweden

**DOI:** 10.3389/fpsyt.2023.1152286

**Published:** 2023-04-24

**Authors:** Katalin Gémes, Heidi Taipale, Emma Björkenstam, Syed Rahman, Klas Gustafsson, Antti Tanskanen, Lisa Ekselius, Ellenor Mittendorfer-Rutz, Magnus Helgesson

**Affiliations:** ^1^Department of Clinical Neuroscience, Division of Insurance Medicine, Karolinska Institutet, Solna, Stockholm, Sweden; ^2^Niuvanniemi Hospital, Kuopio, Finland; ^3^School of Pharmacy, University of Eastern Finland, Kuopio, Finland; ^4^Department of Medical Sciences, Psychiatry, Uppsala University, Uppsala, Sweden; ^5^Department of Women’s and Children’s Health, Uppsala University, Uppsala, Sweden; ^6^Department of Public Health and Caring Sciences, Health Equity and Working Life, Uppsala University, Uppsala, Sweden

**Keywords:** attention deficit and hyperactivity disorder, medication use, population-based, initiation, discontinuation

## Abstract

**Introduction:**

Long-term medication use is a recommended treatment for attention-deficit/hyperactivity disorder (ADHD), however, discontinuation is common. Non-medical factors which might influence initiation and discontinuation are understudied. Therefore, we investigated how different sociodemographic factors and comorbidities were associated with the initiation and discontinuation of ADHD medication use among young adults.

**Methods and results:**

We conducted a population-based prospective cohort study using individually linked administrative register data, in which we included all individuals residing in Sweden, between the age of 19 and 29 who were first diagnosed with ADHD between January 2006 and December 2016 (*n* = 59224). ADHD medication initiation was defined as the first prescription of ADHD medication in the period from 3 months before to 6 months after the cohort entry date. Those who initiated ADHD medication were followed up for medication use until discontinuation, death/emigration, or until 2019. Logistic and Cox regression models were used to investigate the associations between sociodemographics, health-related predictors and initiation, as well as discontinuation. Overall, 48.7% of the 41399 individuals initiated ADHD medication, most often methylphenidate (87%). Among the initiators, 15462 (77%) discontinued medication use during the follow-up (median time: 150 days). After mutually adjusting all other predictors, initiation was positively associated with older age, male sex, higher level of education, and negatively associated with living at home with parents, immigrant status, being unemployed during the year before inclusion, being on disability pension, having autism, substance use, schizophrenia-spectrum disorders, other mental disability/developmental disorders, cardiovascular diseases or previous accidents. Discontinuation was positively associated with being born abroad, living in big cities, being unemployed during the year before inclusion, having cancer, and negatively associated with a higher educational level, having depression, anxiety or stress-related disorder, autism spectrum disorder or diabetes.

**Conclusion:**

Besides medical factors, sociodemographics, such as educational attainment and immigrant status might also play a role in the initiation and discontinuation of ADHD medication use among young adults newly diagnosed with ADHD.

## Introduction

Attention-deficit/hyperactivity disorder (ADHD) is a common neuropsychiatric functional disorder, including symptoms such as attention deficiencies, problems with controlling the activity level, and impulsiveness that might affect social life, educational attainment, and workability negatively (1–4). The estimated prevalence of ADHD is 3–5% among adults in high-income countries (5), and the number of diagnosed cases among young adults is increasing (6–8).

The usual and recommended treatment for ADHD is multimodal treatment with or without the use of ADHD-related medications to facilitate everyday functioning both in the family and at work or school (9, 10). In Sweden, four stimulants, i.e., methylphenidate, lisdexamfetamine, dexamphetamine, and amphetamine, and one non-stimulant, i.e., atomoxetine (together with guanfacine) are the five main active substances prescribed for patients with ADHD with a primary choice of methylphenidate, but dexamphetamine and guanfacine are only approved for use among children and teenagers (11, 12). Based on individual needs, medication can be used in combination, such as stimulants together with non-stimulant, and in different forms, such as extended-release forms. Evidence from clinical trials in adults shows a good short-term effect of ADHD medication on improving functional ability through relieving core symptoms of ADHD such as difficulties in concentration, planning, organizing, and regulating emotions (1, 7, 13, 14). The effect of ADHD medication on long-term outcomes is less researched, but some studies suggest that it might improve symptoms (11, 15), and decrease the risk of unemployment (16) and unintentional injuries (17).

In general, stimulants seem to be more efficacious to alleviate symptoms of ADHD than non-stimulants, but the side effects of stimulants can hinder tolerability in some individuals (11, 18, 19). While adherence to ADHD medication is important to achieve the desired treatment outcomes, discontinuation is common regardless of pharmacological treatment options (20) but varies by the form and types of medication (14, 19). A study on ADHD medication adherence between 2006 and 2009 in Sweden found that in 3 years 50% of individuals discontinued their medication use (21). However, it is less known besides individual variation of tolerability of ADHD medication, what role socioeconomic, work-and health-related factors have in the initiation, discontinuation, and long-term adherence to ADHD medication.

Among children, low socioeconomic status, social adversities, and mental comorbidities were associated with a higher probability of ADHD medication initiation (22–25). Furthermore, low socioeconomic status and comorbid mental disorders have also been associated with lower adherence to ADHD medication (15, 26). There is, however, less research on the association between different patient-related factors such as sociodemographic, work-and health-related factors and comorbidities and ADHD medication adherence among young adults diagnosed with ADHD. Previous studies were conducted either in smaller samples or in selected populations (26–29). As underdiagnosis and undertreatment among young adults with ADHD can lead to difficulties at work, during education or in family relations, and contribute to the development of comorbid mental disorders (30), we intend to address the current knowledge gaps and investigate predictors of initiation and discontinuation of ADHD medication in a population-based cohort of young adults using administrative register data with nationwide coverage.

We aimed to investigate how different sociodemographic, work-and health-related factors and comorbidities were associated with the initiation and discontinuation of ADHD medication use among young adults.

## Methods

### Study population

**Figure 1:**
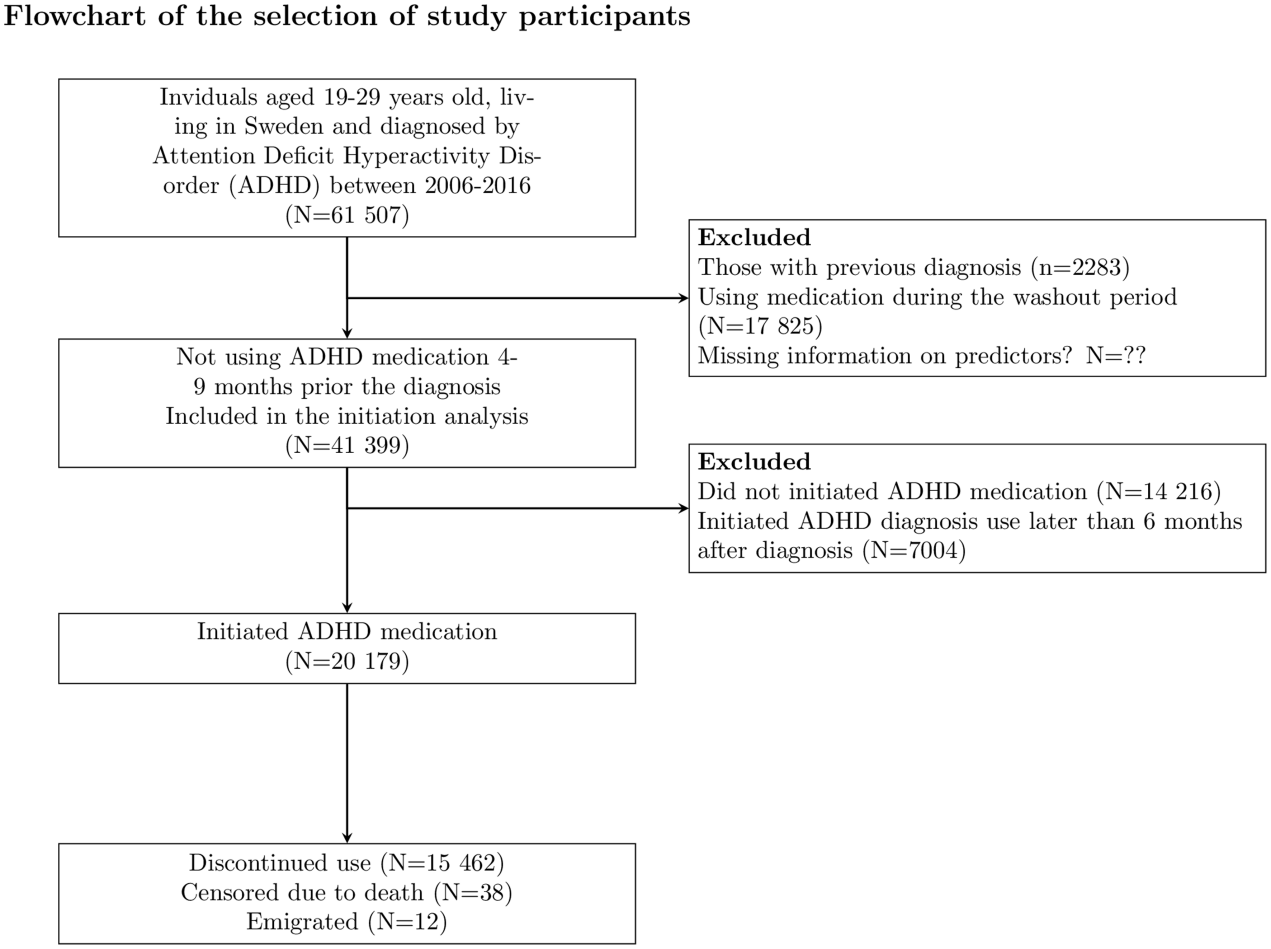
Selection of study participants.

In this register-based study, all individuals residing in Sweden, who were between the age of 19 and 29 and first diagnosed with ADHD between January 2006 and December 2016 (*N* = 61507) were included (Figure 1). The date of the diagnosis of ADHD was considered to be the cohort entry date. ADHD diagnosis was defined from inpatient or specialized outpatient care records by the International Classification of Diseases, Version 10 (ICD-10) (31) as code F90. Patients who were using ADHD medication already 4–9 months before diagnosis were excluded (Figure 1).

### Data source

Individual-level microdata from administrative national registers were linked through the unique (de-identified) personal identification number (32). Inpatient and specialized outpatient care data from the National Patient Register were used to identify ADHD and comorbidities by ICD-10 codes (33). Purchased medication data were obtained from the Prescribed Drug Register, which covers 99% of prescribed drug expenditures in pharmacies in Sweden, using Anatomical-Therapeutic-Chemical (ATC) classification codes (34). Dates of death were obtained from the Cause of Death Register (35). These registers are held by the National Board of Health and Welfare. Sociodemographic and labor market data were derived from the Longitudinal Database for Health Insurance and Labor Market Studies (LISA) held by Statistic Sweden (36). Information on length and grade of sickness absence spells longer than 14 days and disability pension were obtained from the Microdata for Analyses of Social Insurance (MiDAS), from the Swedish Social Insurance Agency (37).

### Outcome measures

All purchases of dispensed ADHD medications by the following ATC codes were included: centrally acting sympathomimetics (N06BA), clonidine (C02AC01), and guanfacine (C02AC02). These medications have been used as a treatment for ADHD internationally (38), and except for clonidine, all are registered as treatment options for ADHD in Sweden. However, to cover the whole range of medication and to be sure that we captured all medications that can be used in ADHD treatment, we also included clonidine. Drug use periods were modeled by the PRE2DUP method by calculating sliding daily dose averages in defined daily doses for each specific drug (39).

*Initiation of ADHD medication* was defined as the first prescription in the period from 3 months before to 6 months after the cohort entry date, and individuals were categorized as initiators vs. non-initiators. Those who initiated ADHD medication were followed up for persistence of ADHD drug use until *discontinuation of use* (second outcome measure), by censoring to death or emigration, 2 years after initiation and end of data linkage on December 31, 2016. We defined discontinuation of ADHD medication use if the drug use period ended before the end of the follow-up. Polytherapy was defined as initiating more than one ADHD drug at the same time.

### Predictors

Several sociodemographic, work-and health-related factors and comorbidities were considered as possible predictors of initiation and discontinuation of ADHD medication use. Sociodemographic factors were assessed at the end of the year preceding the ADHD diagnosis and included level of education [low (≤9 years); medium (10–12 years); high-level education (≥13)], family situation (married/cohabiting and living without children; married/cohabiting and living with children; single/divorced/separated/widowed and living without children; single/divorced/separated/widowed and living with children; age younger than 20 years old and living with parents), country of birth (Sweden; other than Sweden), living region (big cities; medium-sized cities; small cities/villages). The following work and health-related factors were considered: being unemployed the year before the ADHD diagnosis (no; 1–180 days; >180 days), having sickness absence allowance over continuous 14 days periods (no; 14–90 days; >90 days) or being on disability pension at the time of the diagnosis. Psychiatric comorbidities were identified by ICD-10 codes from specialized outpatient and inpatient health within 2 years before the cohort entry date: depression/bipolar disorder (F30-34); anxiety and stress-related disorders (F40-48); autism-spectrum disorders (F84); substance use disorders (F10-19); behavioral and emotional disorders, excluding ADHD (F91-98); mental disability/developmental disorders (F70-89); schizophrenia-spectrum disorders (F20-29); other mental disorders (other F codes). Somatic comorbidities were categorized as musculoskeletal disorder (M00-M99); asthma (J45); diabetes (E10-E11); cancer (C00-D48); cardiovascular diseases (I00-I99); accidents (S00-S99); and other somatic disorders (other codes except O.80 and Z00-99).

### Statistical analyses

The distributions of study variables for the total population and by initiation and discontinuation are presented as a mean value and the standard deviation for continuous variables, and the number of individuals and percentages for categorical variables. Logistic regression was used to analyze the association between each predictor and the initiation of ADHD medication (the reference category was “non-initiation”). Odds ratios (ORs) with 95% confidence intervals (CIs) were calculated for the crude model and by adjusting to the other predictors simultaneously. Cox proportional hazard regression was used to estimate the hazard of discontinuation of medication use compared with continuous use during the follow-up. Crude and multi-adjusted hazard ratios (HR) and 95% CIs were calculated.

## Results

### Initiation

Of the 59224 individuals with the first ADHD diagnosis, 41399 did not use medication during 4–9 months before diagnosis and were therefore included in this study (Figure 1). The distribution of study variables for these individuals is presented in Table 1. Among them, 20179 individuals (48.7%) initiated any ADHD medication use between 3 months before and 6 months after the date of the diagnosis (Figure 1). The majority (87%) of them initiated methylphenidate, 7.2% initiated atomoxetine, and 3.8% initiated lisdexamfetamine (Table 2; Supplementary Table S1). Overall 14216 (34.3%) individuals did not initiate ADHD medication during the entire follow-up and 7004 (16.9%) initiated medication later than 6 months after the date of ADHD diagnosis.

**Table 1:** Characteristics of study participants (*N* = 41399).

Mean age (SD)	21.5 (3.2)
Male sex % (*N*)	55.4 (22940)
*Level of education % (N)*	
Compulsory (≤9 years)	54.4 (22518)
Secondary (10–12 years)	33.6 (13918)
University, college (≥13 years)	6.2 (2578)
Unknown	5.8 (2385)
*Family situation % (N)*	
Married/living with partner without children	0.8 (312)
Married/living with partner with children	5.0 (2059)
Single/divorced/separated/widowed without children	52.6 (21776)
Single/divorced/separated/widowed with children	3.6 (1473)
Child (<20 years old) and living with parents	38.1 (15779)
*Country of birth % (N)*	
Sweden	92.8 (38436)
Other	7.2 (2963)
*Type of living area % (N)*	
Large cities	37.1 (15346)
Medium-sized cities	34.8 (14401)
Small cities/villages/rural area	28.2 (11652)
*Unemployment during the previous year % (N)*	
No unemployment	70.4 (29137)
1–180 days	26.2 (10830)
180 days	3.5 (1432)
*Sickness absence during the previous year % (N)*	
<14 days	88.7 (36717)
14–90 days	5.2 (2149)
>90 days	6.1 (2533)
On disability pension % (*N*)	20.2 (8361)
*Psychiatric comorbidities % (N)*	
Depression or bipolar disorder	21.7 (9000)
Anxiety and stress-related disorders	27.6 (11426)
Autism-spectrum disorder	10.4 (4306)
Substance abuse	13.5 (5598)
Behavioral and emotional disorders	3.1 (1275)
Mental disability/developmental disorders	4.1 (1690)
Schizophrenia/psychoses	1.7 (699)
Other mental disorders	12.2 (5058)
*Somatic co-morbidities % (N)*	
Musculoskeletal disorders	5.7 (2365)
Asthma	1.3 (539)
Diabetes	1.0 (419)
Cancer	1.4 (570)
Cardiovascular disease	1.2 (476)
Accidents	12.2 (5033)
Other somatic disorders	39.0 (16135)
*Other medication use % (N)*	
Antidepressant	37.0 (15336)
Hypnotic	23.4 (9667)
Anxiolytic	22.1 (9147)
Antipsychotic	10.9 (4497)

SD, standard deviation.

**Table 2:** Distribution of predictors by associated with initiating (vs. not initiating) and discontinuing medication use among study participants use.

	Not initiating *N* = 21220	Initiator *N* = 20179	Not discontinued *N* = 4717	Discontinued *N* = 15462
Mean age (SD)	21.0 (3.1)	22.0 (3.2)	22.8 (3.2)	21.8 (3.2)
%*N*				
Men	41.9 (8899)	47.4 (9560)	47.4 (1391)	47.4 (8169)
Level of education				
Unknown	9.0 (1901)	2.4 (484)	1.5 (45)	2.6 (439)
Compulsory	58.8 (12469)	49.8 (10049)	35.6 (1045)	52.2 (9004)
Secondary	28.1 (5958)	39.5 (7960)	49.3 (1447)	37.8 (6513)
University, college	4.2 (892)	8.4 (1686)	13.5 (396)	7.5 (1290)
*Family situation*				
Married/living with partner without children	0.7 (146)	0.8 (166)	0.9 (43)	0.8 (123)
Married/living with partner with children	3.9 (818)	6.2 (1241)	7.8 (367)	5.7 (874)
Single/divorced/separated/widowed without children	48.1 (10203)	57.4 (11573)	63.8 (3011)	55.4 (8562)
Single/divorced/separated/widowed with children	2.9 (624)	4.2 (849)	4.7 (223)	4.1 (626)
Child (<20 years old) living at home	44.4 (9429)	31.5 (6350)	22.8 (1073)	34.1 (5277)
*Country of birth*				
Sweden	92.3 (19575)	93.5 (18861)	93.9 (4431)	93.3 (14430)
Other	7.8 (1645)	6.5 (1312)	6.1 (286)	6.7 (1032)
*Living region*				
Big cities	33.4 (7092)	40.9 (8255)	40.0 (1887)	41.2 (6367)
Medium-sized cities	37.7 (7996)	31.7 (6405)	31.7 (1494)	31.8 (4911)
Small cities/villages	28.9 (6132)	27.4 (5520)	28.3 (1336)	27.1 (4184)
*Unemployment during the previous year*				
No	70.7 (15010)	70.0 (14127)	71.3 (3361)	69.6 (10766)
1–180 days	25.8 (5478)	26.5 (5352)	25.0 (1179)	27.0 (4173)
>180 days	3.5 (732)	3.5 (700)	3.8 (177)	3.4 (523)
*Sickness absence during the previous year*				
No	90.6 (19215)	86.7 (17502)	83.5 (3937)	87.7 (13565)
1–90 days	4.2 (882)	6.3 (1267)	7.6 (357)	5.9 (910)
>90 days	5.3 (1123)	7.0 (1410)	9.0 (423)	6.4 (987)
On disability pension at baseline	29.4 (6243)	10.5 (2118)	10.3 (485)	10.6 (1633)
*Mental comorbidities*
Depression or bipolar disorder	20.8 (4423)	22.7 (4577)	25.2 (1189)	21.9 (3388)
Anxiety and stress-related disorders	27.2 (5767)	28.0 (5659)	31.5 (1486)	27.0 (4173)
Autism-spectrum disorder	11.9 (2521)	8.9 (1785)	10.0 (470)	8.5 (1315)
Substance abuse	14.4 (3052)	12.6 (2546)	11.7 (552)	12.9 (1994)
Behavioral and emotional disorders	3.3 (691)	2.9 (584)	2.7 (126)	3.0 (458)
Mental disability/developmental disorders	5.3 (1126)	2.8 (564)	2.5 (118)	2.9 (446)
Schizophrenia/psychoses	2.2 (458)	1.2 (241)	1.1 (53)	1.2 (188)
Other mental disorders	12.0 (2546)	12.5 (2512)	13.7 (646)	12.1 (1866)
*Somatic comorbidities*				
Musculoskeletal disorders	5.5 (1173)	5.9 (1192)	6.3 (298)	5.8 (894)
Asthma	1.4 (286)	1.3 (253)	1.3 (61)	1.2 (192)
Diabetes	1.1 (228)	1.0 (191)	1.2 (55)	0.9 (136)
Cancer	1.3 (273)	1.5 (297)	1.2 (56)	1.6 (241)
Cardiovascular disease	1.4 (291)	0.9 (185)	0.9 (41)	0.9 (144)
Accidents	12.8 (2707)	11.5 (2326)	11.6 (548)	11.5 (1778)
Other somatic disorders	39.1 (8290)	38.9 (7845)	39.6 (1869)	38.7 (5976)
*Initial ADHD medication*				
Methylphenidate	Not applicable		87.0 (4104)	82.1 (12697)
Atomoxetine	Not applicable		7.2 (338)	13.9 (2146)
Lisdexamfetamine	Not applicable		3.8 (179)	2.5 (387)
Other	Not applicable		0.7 (33)	0.6 (89)
Polytherapy	Not applicable		1.3 (63)	0.9 (143)

SD, standard deviation.

**Figure 2:**
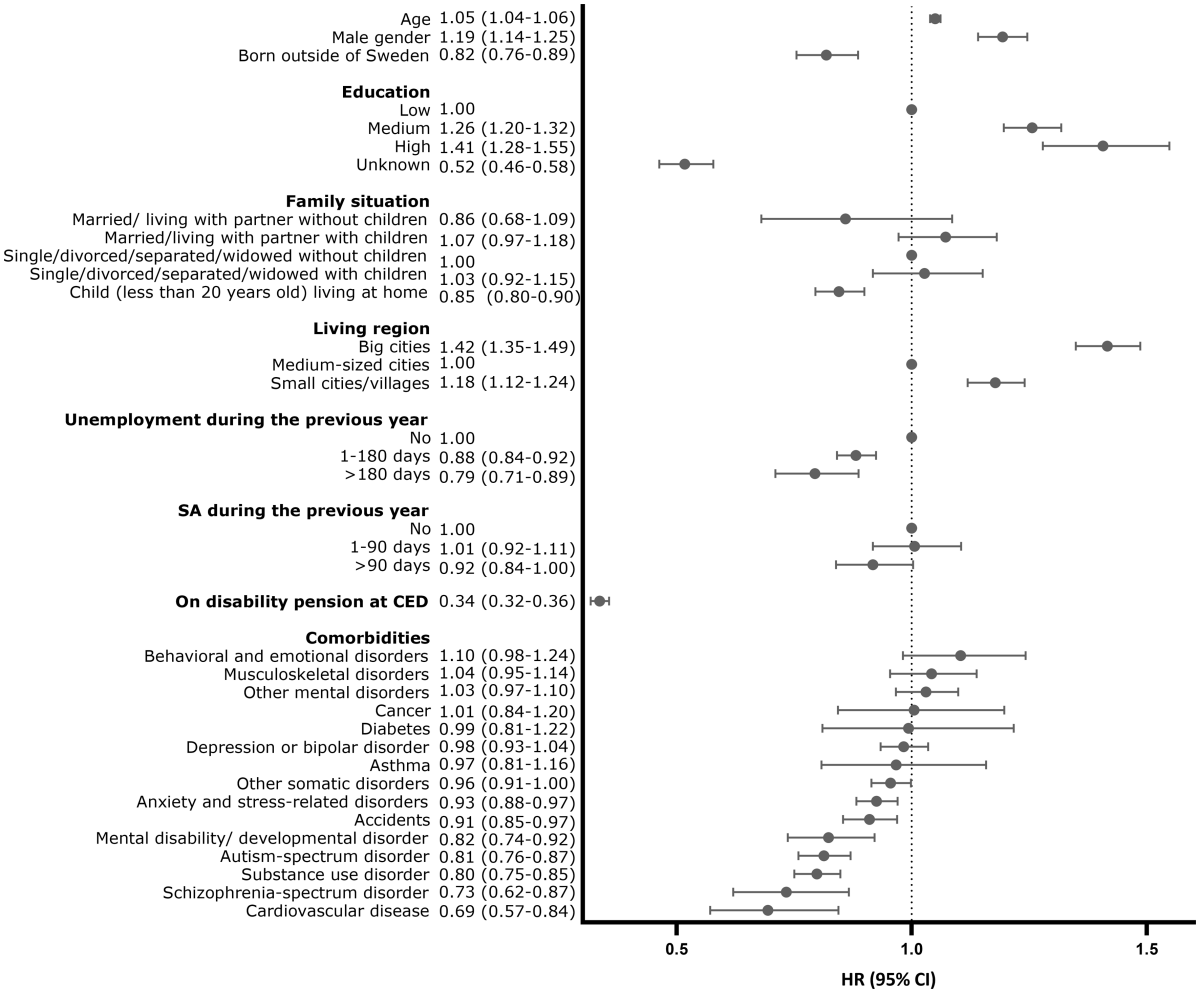
Associations between predictors and initiation of medication use for attention deficit hyperactivity disorder (odds ratios and 95% confidence intervals comparing initiators with non-initiators).

The distribution of predictors by initiation is presented in Table 2. In the unadjusted model, age, sex, education, family situation, country of birth, living region, sickness absence during the previous year, and disability pension at baseline as well as comorbid mental and somatic disorders were associated with initiation. After mutually adjusting for all other predictors, older age, male sex, higher level of education, living in larger cities or smaller cities, villages compared with medium-sized cities were positively associated with the initiation of ADHD medication (Figure 2; Supplementary Table S2). Being <20 years old and living at home with parents, being born abroad, having unemployment days during the previous year, being on disability pension, having comorbid mental disorders, e.g., autism, substance use, schizophrenia-spectrum disorders, other mental disability/developmental disorders, or comorbid somatic diseases, e.g., cardiovascular diseases or accidents were negatively associated with the initiation of ADHD medication in the multivariate analysis (Figure 2; Supplementary Table S2).

### Discontinuation

**Figure 3:**
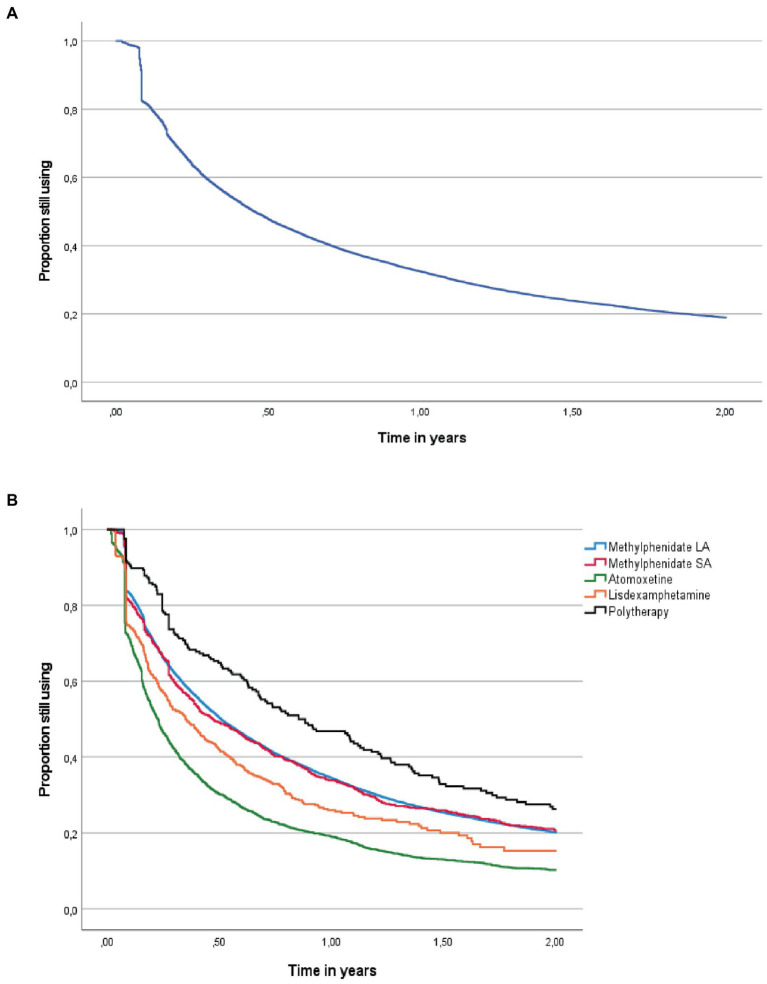
**(A)** Time to discontinuation of attention deficit hyperactivity disorder medication use (*N* = 20179). **(B)** Time to discontinuation of use for 5 most common initial attention deficit hyperactivity disorder medication (other medications have very few users). LA, long-acting; SA, short-acting; polytherapy, initiation with two or more drugs at the same time.

**Figure 4:**
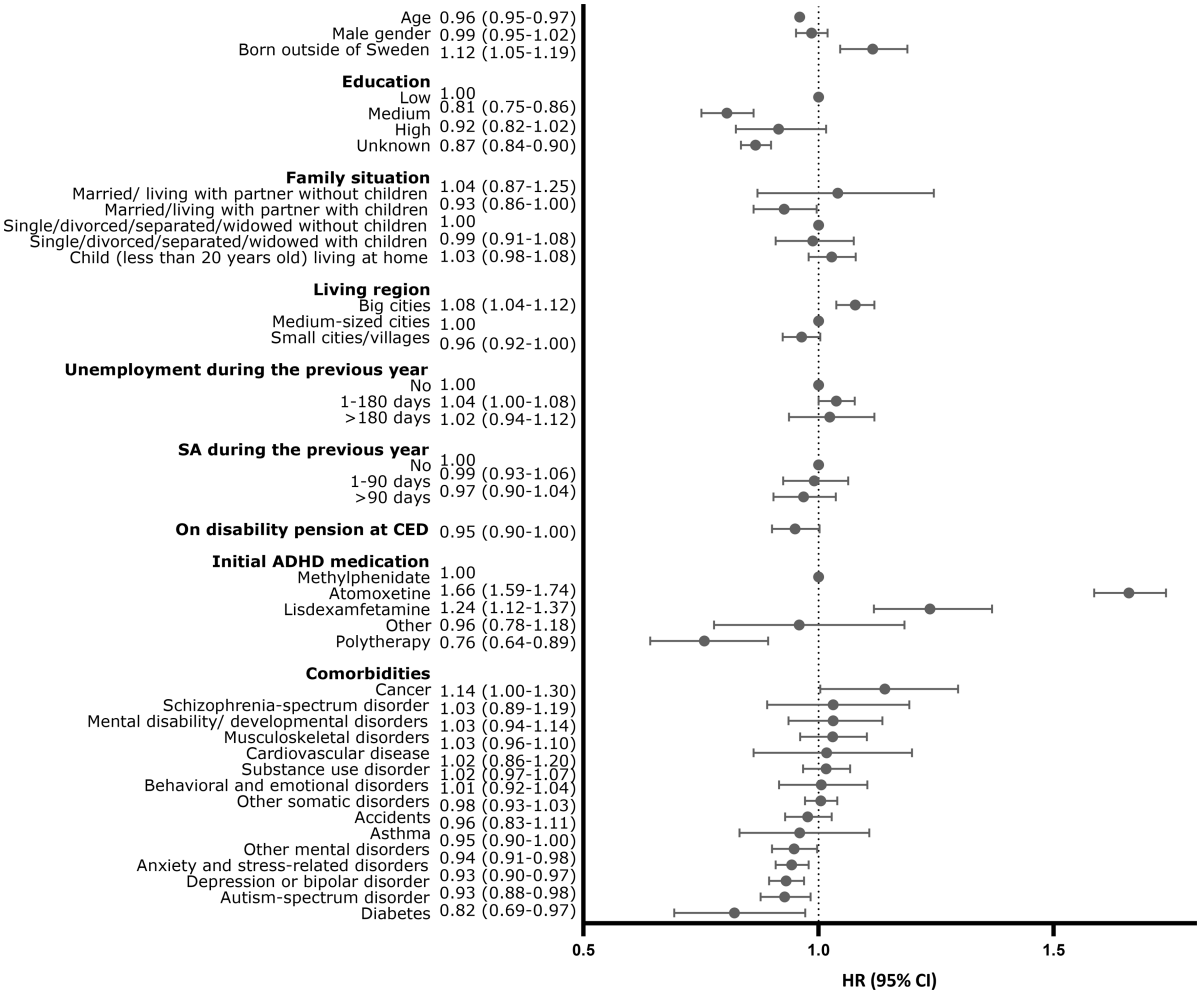
Associations between predictors and discontinuation of attention deficit hyperactivity disorder (ADHD) medication (hazard ratios and 95% confidence intervals comparing those who discontinued with those who continued ADHD during the follow-up.

Among those who initiated ADHD medication, 15462 (76.6%) discontinued medication use during the follow-up. Overall, 38 individuals died, and 13 individuals emigrated during the follow-up (Figures 1, 3B). The median person-time of first ADHD medication use during the follow-up was 150 days (interquartile range (IQR): 57–425 days). The median time of medication use among those who discontinued was 124 days (IQR 48–342), 60.1% (*N* = 10358) had discontinued within 6 months and 76.4% (*N* = 13180) had discontinued within 1 year. The time to discontinuation is presented in Figures 3A, B. Of those who discontinued use, 66.8% (*n* = 10325) re-initiated use later during the follow-up. The median time to reinitiation was 91 days (IQR: 43–258). For further analysis, long-acting and short-acting methylphenidate use were combined, as they followed similar discontinuation patterns. The distribution of study variables by discontinuation among those who initiated ADHD medication and the associations between the predictors and discontinuation are shown in Table 2, Figure 4, and Supplementary Table S2, respectively.

In the crude analysis, age, educational level, family situation, country of birth, sickness absence in the previous year, mental and somatic comorbidities, and type of medication were associated with discontinuation. In the multivariate analysis being born abroad, living in big cities, being unemployed during the previous year, having cancer, and initiating atomoxetine or lisdexamfetamine compared with methylphenidate was positively associated with medication discontinuation. A higher educational level, depression, anxiety or stress-related disorder, autism spectrum disorder or diabetes were negatively associated with ADHD medication discontinuation. Of those who discontinued ADHD medication use during the follow-up (*N* = 15462), 66.8% (*N* = 10325) re-initiated medication use later during the follow-up. The mean time to re-initiation was 245 days (SD 394), median time was 91 days (IQR 43–258).

## Discussion

### Summary of The findings

In this population-based study, 48.7% of young adults, who were diagnosed with ADHD for the first time in adult age started medication 3 months before and 6 months after the diagnosis of ADHD. Among those who started medication, 76.6% discontinued the first choice of medication within the first 2 years. Initiation and discontinuation of ADHD medication use were associated with different sociodemographic and clinical characteristics. Older age, higher educational level, and being born in Sweden were associated with a higher chance of initiation and a lower chance of discontinuation. Female sex, living in medium-sized cities, being <20 years and living at home, being on disability pension or being unemployed, and having comorbid mental disorders or cardiovascular disorders were associated with a lower probability of initiation of ADHD medication independent from the other predictors. Family situation, comorbid mental or somatic disorders, and type of medication were independent predictors for medication discontinuation.

### Comparison with previous studies

There are only a few studies that investigated predictors of ADHD medication use in adults, most of the previous studies were conducted among children and adolescents. The rate of initiation of medication was similar to those observed in children and adolescents (40).

Concerning initiation, the most comprehensive register-based study conducted in the United States among individuals younger than 21 years old found similar results to our study in which male sex and higher socioeconomic status were positively associated with ADHD medication initiation (24). A United Kingdom study, including children and young individuals, described a social gradient in the initiation of ADHD medication, with lower odds of ADHD medication initiation among those with social deprivation (22). While we did not measure social gradient, we found that educational level, which is in strong correlation with social status, was one of the strongest predictors of initiation of ADHD medication. Besides education, being born abroad was negatively associated with ADHD medication initialization in our study. A study among refugee minors in Sweden also reported lower use of ADHD medication among non-accompanied refugees compared with Swedish-born (41). While the two studies cannot be compared directly, both of them suggested a lower initiation of ADHD medication within the immigrant population. Immigrants in general might be prone to underutilizing psychiatric health care (41–44), but other factors such as cultural beliefs and attitudes toward behavioral symptoms and medication of ADHD (45, 46), health literacy (47), language problems, or occupation might also contribute to less frequent medication initiation. A study among children, who were born between 1995 and 2004 in Sweden, found that children who had only foreign-born parents were less likely to use medication compared with children who had at least one Swedish-born parent, suggesting that ADHD medication use might be influenced by cultural factors (46).

Similar to our findings among adults, mental comorbidities were associated with a lower likelihood of ADHD treatment initiation among youth (24) and children (29). Mental comorbidities might be a barrier to ADHD medication initiation due to the possible drug interactions between ADHD medication and other psychotropics (48, 49). Treatment guidelines for ADHD with comorbid mental disorders, such as psychosis, bipolar disorder, substance abuse, severe depression, and anxiety recommend treating the more severe condition first in the case of contraindication of ADHD medication with other psychotropics (11, 50). For example, there are some safety concerns about the co-medication of drugs used in ADHD with antipsychotics (51) and with some antidepressants (50). Furthermore, there are some concerns, that using stimulants in comorbid substance use disorders might increase the risk of relapse (18), however, there are also results showing that methylphenidate and lisdexamphetamine use might prevent substance use episodes in ADHD patients (52) or hospitalization and all-cause mortality among individuals with amphetamine use disorder (53), and untreated ADHD itself is a risk factor for future substance use disorder (50).

There were only a few studies that investigated ADHD medication adherence and discontinuation in adults and they described a similarly high rate of discontinuation similar to ours (29, 54, 55). In our study, the vast majority of individuals with newly diagnosed ADHD initiated methylphenidate, as methylphenidate is the recommended first-line medication for ADHD in Sweden (11) due to the best balance in effectiveness and side effects (50). We found that methylphenidate initiation was associated with a lower likelihood of discontinuation compared with other medications, which is reassuring, concerning the pharmacological characteristics of methylphenidate, as effectiveness and side effects of ADHD medication have been described as the strongest predictors of adherence and discontinuation (29). The higher risk of discontinuation of atomoxetine might be explained by the described common side effects, such as loss of appetite, nausea, sleep problems, dry mouth, erectile dysfunction, and headaches which might explain the lower tolerability compared with the other medications, but also may be due to the described lower efficacy of atomoxetine to alleviate ADHD symptoms (15, 19, 56).

Concerning sociodemographic factors, we found that lower educational level, and country of birth but not male sex were predictors of discontinuation. Among adolescents and young individuals, male sex and higher education were associated with better adherence to ADHD treatment in a United States register-based study (24). A current review of factors influencing ADHD medication adherence in adults concluded that sex, younger age, and lower education were the most common factors that were associated with both early and later phases of medication discontinuation (29). Concerning sex, both female and male sex predicted medication discontinuation in different studies (29), and some studies reported no medication use difference between women and men (57). This heterogeneity of results might be explained partly due to the different definitions of medication discontinuation and adherence (27) but also differences in study design, study population, or the different socioeconomic environments, healthcare, and drug reimbursement practices might contribute to this inconsistency (58). Therefore, the role of social and economic factors and cultural differences in adherence to ADHD medication needs more research, especially as they might be the possible starting point for developing interventions such as targeted patient education, that have been suggested to improve medication adherence among individuals with ADHD (29).

Concerning mental comorbidities, similar to our results, some studies also described a lower risk of ADHD medication discontinuation among adults with mental comorbidities compared to those without (29). Among children, psychiatric comorbidities and concomitant use of psychiatric medication were also described as factors that were associated with higher adherence (29). The reason for these findings is not clear, but ADHD symptoms may make it difficult for patients to keep a consistent medication regime and a good daily routine (50), whereas patients with other mental comorbidities may have already developed better compliance with medication use through previous healthcare contacts, psychoeducation and therapies (29).

### Strengths and limitations

The major strength of this study was that it covers the whole population of Sweden regardless of socioeconomic or health status or other factors. All residents, who have been in contact with secondary health care due to ADHD are captured, therefore the results are representative of the whole Swedish population, and the large sample size allows us to investigate a wide range of sociodemographic and health-related predictors. However, ADHD is often diagnosed by the DSM IV and DSM-5 diagnostic criteria which limitedly resemble the diagnostic criteria of ICD-10 which is registered in the National Patient Register (59). Another strength of our study is that the use of high-quality information from administrative registers ascertained a lower likelihood of misclassification on medication use, sociodemographic, and health-related information better than self-reported information. On the other hand, there might be some possible important predictors that were not available in our data, such as initiation of psychotherapies, symptoms of ADHD, or reported side effects of medication use, which might lead to medication discontinuation. Another limitation is that the Prescribed Drug Register only includes information on dispensed medication, therefore we had no information on prescribed but not dispensed medications (i.e., primary non-adherence). Furthermore, we had no reliable information for which indication the medication was prescribed, therefore we only included those medications in our analysis which are among the recommended ones and registered for ADHD in Sweden. Off-label use for ADHD of other medications, for example, bupropion, were therefore not included among the ADHD medications, however, it is unlikely that this would influence the results considerably. Furthermore, information on comorbidities was accessed through the National Patient Register, therefore comorbidities diagnosed only in primary health care were not identified. Finally, our results can only be generalized for individuals diagnosed with ADHD in young adulthood. Individuals with childhood ADHD probably have experienced more severe symptoms, are more likely to have other comorbidities associated with worse prognosis (60), and might be more hindered to achieve a higher level of education. Our study results can have limited generalizability to countries with different social insurance and health care systems or in different stages of economic development, such as countries with fewer resources, less equal access to health care services or higher rates of medication co-payment. Therefore, socioeconomic differences might play a much larger role in such countries for starting and maintaining medication use among individuals with ADHD compared with Sweden.

## Conclusion

The results of this population-based study suggest that besides medical factors, sociodemographics, such as educational attainment and country of birth might also play a role in the initiation and discontinuation of ADHD medication use among young adults newly diagnosed with ADHD. Future studies need to focus on understanding the mechanism behind these differences in medication initiation and discontinuation as a proxy of medication adherence and identify patient groups who require extra support for achieving treatment targets.

## Data availability statement

The data analyzed in this study is subject to the following licenses/restrictions: The project utilized data from the REWHARD consortium, supported by the Swedish Research Council (VR grant number. 2017-00624). These data cannot be made publicly available due to privacy regulations. According to the General Data Protection Regulation, the Swedish law SFS 2018:218, the Swedish Data Protection Act, the Swedish Ethical Review Act, and the Public Access to Information and Secrecy Act, these types of sensitive data can only be made available for specific purposes, including research, that meet the criteria for access to these type of sensitive and confidential data as determined by a legal review. Requests to access these datasets should be directed to Kristina Alexanderson (kristina.alexanderson@ki.se).

## Ethics statement

This project was evaluated and approved by the regional ethical review board in Stockholm, Sweden. The ethical committee approval number is 2007/762-31. The ethical review board approved the study and waived the requirement that informed consent of research subjects should be obtained. Written informed consent for participation was not required for this study in accordance with the national legislation and the institutional requirements.

## Author contributions

KGé, HT, EB, SR, KGu, AT, LE, EM-R, and MH contributed to the conceptualization and design of the study, the interpretation of the results, the critical revision of the manuscript draft. MH provided funding for the research. HT run the analysis. KGé drafted the manuscript. All authors approved the publication content and agree to be accountable for all aspects of the work in ensuring that questions related to the accuracy or integrity of any part of the work are appropriately investigated and resolved.

## Funding

This work was financially supported by a research grant from AFA Försäkring (grant number 180295, receiver: MH). We utilized data from the REWHARD consortium supported by the Swedish Research Council (grant number 2017-00624). There was no additional external funding received for this study. The funders had no role in study design, data collection and analysis, decision to publish, or preparation of the manuscript.

## Conflict of interest

The authors declare that the research was conducted in the absence of any commercial or financial relationships that could be construed as a potential conflict of interest.

## Publisher’s note

All claims expressed in this article are solely those of the authors and do not necessarily represent those of their affiliated organizations, or those of the publisher, the editors and the reviewers. Any product that may be evaluated in this article, or claim that may be made by its manufacturer, is not guaranteed or endorsed by the publisher.

## Supplementary material

The Supplementary material for this article can be found online at: https://www.frontiersin.org/articles/10.3389/fpsyt.2023.1152286/full#supplementary-material
