# Supplementary material for: The role of sociodemographic and clinical factors in the initiation and discontinuation of attention deficit hyperactivity disorder medication among young adults in Sweden
Source: Front Psychiatry. 2023 Apr 24;14:1152286. doi: 10.3389/fpsyt.2023.1152286 (PMC10165120; doi:10.3389/fpsyt.2023.1152286)
Supplement: Supplementary file 1 [file Table_1.DOCX]

**Table S1**: Distribution of initiators by type of medication

| **Medication** | **N of individuals** | **Percentage (%)** |
| --- | --- | --- |
| *Clonidine* | 14 | 7 |
| *Guanfacine* | 4 | 2 |
| *Amfetamine* | 7 | 3 |
| *Dexamfetamine* | 27 | 13 |
| *Methylphenidate long-acting* | 15,995 | 79 |
| *Methylphenidate short-lasting* | 806 | 4 |
| *Modafinil* | 70 | 35 |
| *Atomoxetin* | 2484 | 12 |
| *Lisdexamfetamin* | 566 | 3 |
| *Polytherapy^*^* | 206 | 1 |

*Initiating more than one type of medication at the same time

| **Table S2.** Association between predictors and initiating compared with not initiating medication use for attention deficit hyperactivity disorder (ADHD) | | | | |  |  |  |  |
| --- | --- | --- | --- | --- | --- | --- | --- | --- |
|  | Initiating medication, among young individuals diagnosed by ADHD (n=41,399) | | Discontinuing ADHD medication use, among initiators (n=20,179) | |  |  |  |  |
|  | Crude OR (95% CI) | Adjusted OR (95% CI)* | Crude HR (95% CI) | Adjusted HR (95% CI)* |  |  |  |  |
| Age | 1.10 (1.10-1.11) | 1.05 (1.04-1.06) | 0.95 (0.94-0.95) | 0.96 (0.95-0.97) |  |  |  |  |
| Men | 1.25 (1.20-1.30) | 1.19 (1.14-1.25) | 0.97 (0.94-1.01) | 0.99 (0.95-1.02) |  |  |  |  |
| Level of education |  |  |  |  |  |  |  |  |
| *Low* | reference | reference | reference | reference |  |  |  |  |
| *Medium* | 1.66 (1.59-1.73) | 1.26 (1.20-1.32) | 0.68 (0.64-0.73) | 0.81 (0.75-0.86) |  |  |  |  |
| *High* | 2.35 (2.15-2.55) | 1.41 (1.28-1.55) | 0.97 (0.88-1.07) | 0.92 (0.82-1.02) |  |  |  |  |
| *Unknown* | 0.32 (0.29-0.35) | 0.52 (0.46-0.58) | 0.78 (0.76-0.81) | 0.87 (0.84-0.90) |  |  |  |  |
| Family situation | |  |  |  |  |  |  |  |
| *Single/divorced/separated/widowed without children* | reference | reference | reference | reference |  |  |  |  |
| *Married/living with partner without children* | 1.00 (0.80-1.25) | 0.86 (0.68-1.09) | 0.97 (0.81-1.16) | 1.04 (0.87-1.25) |  |  |  |  |
| *Single/divorced/separated/widowed with children* | 1.20 (1.08-1.34) | 1.03 (0.92-1.15) | 0.96 (0.88-1.04) | 0.99 (0.91-1.08) |  |  |  |  |
| *Married/living with partner with children* | 1.34 (1.22-1.47) | 1.07 (0.97-1.18) | 0.86 (0.81-0.93) | 0.93 (0.86-1.00) |  |  |  |  |
| *Child (less than 20 years old) living at home* | 0.59 (0.57-0.62) | 0.85 (0.80-0.90) | 1.34 (1.29-1.38) | 1.03 (0.98-1.08) |  |  |  |  |
| Country of birth | |  |  |  |  |  |  |  |
| *Sweden* | reference | reference | reference | reference |  |  |  |  |
| *Other* | 0.83 (0.77-0.90) | 0.82 (0.76-0.89) | 1.08 (1.02-1.15) | 1.12 (1.05-1.19) |  |  |  |  |
| Living region | |  |  |  |  |  |  |  |
| *Big cities* | 1.45 (1.39-1.52) | 1.42 (1.35-1.49) | 1.05 (1.01-1.09) | 1.08 (1.04-1.12) |  |  |  |  |
| *Medium-sized cities* | reference | reference | reference | reference |  |  |  |  |
| *Small cities/villages* | 1.12 (1.07-1.18) | 1.18 (1.12-1.24) | 0.98 (0.94-1.02) | 0.96 (0.92-1.00) |  |  |  |  |
| Unemployment during the previous year | | |  |  |  |  |  |  |
| No | reference | reference | reference | reference |  |  |  |  |
| 1-180 days | 1.04 (0.99-1.09) | 0.88 (0.84-0.92) | 1.02 (0.98-1.06) | 1.04 (1.00-1.08) |  |  |  |  |
| >180 days | 1.02 (0.91-1.13) | 0.79 (0.71-0.89) | 0.96 (0.88-1.05) | 1.02 (0.94-1.12) |  |  |  |  |
| SA during the previous year | | |  |  |  |  |  |  |
| No | reference | reference | reference | reference |  |  |  |  |
| 1-90 days | 1.58 (1.44-1.72) | 1.01 (0.92-1.11) | 0.86 (0.80-0.92) | 0.99 (0.93-1.06) |  |  |  |  |
| >90 days | 1.38 (1.27-1.50) | 0.92 (0.84-1.00) | 0.80 (0.75-0.85) | 0.97 (0.90-1.04) |  |  |  |  |
| On disability pension at cohort entry date (ref: not on disability pension) | 0.28 (0.27-0.30) | 0.34 (0.32-0.36) | 1.00 (0.95-1.05) | 0.95 (0.90-1.00) |  |  |  |  |
| Comorbidities |  |  |  |  |  |  |  |  |
| Depression or bipolar disorder | 1.11 (1.06-1.17) | 0.98 (0.93-1.04) | 0.89 (0.86-0.93) | 0.93 (0.90-0.97) |  |  |  |  |
| Anxiety and stress-related disorders | 1.04 (1.00-1.09) | 0.93 (0.88-0.97) | 0.91 (0.87-0.94) | 0.94 (0.91-0.98) |  |  |  |  |
| Autism-spectrum disorder | 0.72 (0.68-0.77) | 0.81 (0.76-0.87) | 0.94 (0.89-1.00) | 0.93 (0.88-0.98) |  |  |  |  |
| Substance use disorder | 0.86 (0.81-0.91) | 0.80 (0.75-0.85) | 1.00 (0.95-1.05) | 1.02 (0.97-1.07) |  |  |  |  |
| Behavioral and emotional disorders | 0.89 (0.79-0.99) | 1.10 (0.98-1.24) | 1.06 (0.96-1.16) | 1.01 (0.92-1.10) |  |  |  |  |
| Mental disability/ developmental disorders | 0.51 (0.46-0.57) | 0.82 (0.74-0.92) | 1.10 (1.00-1.21) | 1.03 (0.94-1.14) |  |  |  |  |
| Schizophrenia/non-affective psychoses | 0.55 (0.47-0.64) | 0.73 (0.62-0.87) | 1.05 (0.91-1.21) | 1.03 (0.89-1.19) |  |  |  |  |
| Other mental disorders | 1.04 (0.98-1.11) | 1.03 (0.97-1.10) | 0.90 (0.86-0.94) | 0.95 (0.90-1.00) |  |  |  |  |
| Musculoskeletal disorders | 1.07 (0.99-1.17) | 1.04 (0.95-1.14) | 0.99 (0.93-1.06) | 1.03 (0.96-1.10) |  |  |  |  |
| Asthma | 0.93 (0.78-1.10) | 0.97 (0.81-1.16) | 0.99 (0.86-1.14) | 0.96 (0.83-1.11) |  |  |  |  |
| Diabetes | 0.88 (0.73-1.07) | 0.99 (0.81-1.22) | 0.84 (0.71-1.00) | 0.82 (0.69-0.97) |  |  |  |  |
| Cancer | 1.15 (0.97-1.35) | 1.01 (0.84-1.20) | 1.09 (0.96-1.24) | 1.14 (1.00-1.30) |  |  |  |  |
| Cardiovascular disease | 0.67 (0.55-0.80) | 0.69 (0.57-0.84) | 0.98 (0.83-1.15) | 1.02 (0.86-1.20) |  |  |  |  |
| Accidents | 0.89 (0.84-0.95) | 0.91 (0.85-0.97) | 1.00 (0.95-1.05) | 0.98 (0.93-1.03) |  |  |  |  |
| Other somatic disorders | 0.99 (0.95-1.03) | 0.96 (0.91-1.00) | 0.99 (0.96-1.02) | 1.01 (0.97-1.04) |  |  |  |  |
| Initial ADHD medication |  |  |  |  |  |  |  |  |
| Methylphenidate | Not relevant | Not relevant | reference | reference |  |  |  |  |
| Atomoxetine | Not relevant | Not relevant | 1.66 (1.59-1.74) | 1.66 (1.59-1.74) |  |  |  |  |
| Lisdexamfetamine | Not relevant | Not relevant | 1.28 (1.15-1.41) | 1.24 (1.12-1.37) |  |  |  |  |
| Other | Not relevant | Not relevant | 0.96 (0.78-1.19) | 0.96 (0.78-1.18) |  |  |  |  |
| Polytherapy | Not relevant | Not relevant | 0.76 (0.64-0.89) | 0.76 (0.64-0.89) |  |  |  |  |
| *Adjusted for all variables listed in this table. | | | | |  |  | 0.99 (0.86-1.14) | 0.96 (0.83-1.11) |
